# Effect of Nitrogen and Aluminum Doping on 3C-SiC Heteroepitaxial Layers Grown on 4° Off-Axis Si (100)

**DOI:** 10.3390/ma14164400

**Published:** 2021-08-06

**Authors:** Cristiano Calabretta, Viviana Scuderi, Ruggero Anzalone, Marco Mauceri, Danilo Crippa, Annalisa Cannizzaro, Simona Boninelli, Francesco La Via

**Affiliations:** 1 Istituto per la Microelettronica e Microsistemi-Consiglio Nazionale delle Ricerche (IMM-CNR), VIII Strada, 5, 95121 Catania, Italy; cristiano.calabretta@imm.cnr.it (C.C.); annalisa.cannizzaro@imm.cnr.it (A.C.); simona.boninelli@imm.cnr.it (S.B.); francesco.lavia@imm.cnr.it (F.L.V.); 2 STMicroelectronics, Stradale Primosole, 50, 95121 Catania, Italy; ruggero.anzalone@st.com; 3 LPE, XVI Strada, 95121 Catania, Italy; Marco.Mauceri@lpe-epi.com; 4 LPE, Via Falzarego, 8, 20021 Baranzate, Italy; Danilo.Crippa@lpe-epi.com; 5 Dipartimento di Fisica e Astronomia, Università di Catania, Via S. Sofia, 64, 95123 Catania, Italy

**Keywords:** 3C-SiC, stacking faults, doping, KOH etching

## Abstract

This work provides a comprehensive investigation of nitrogen and aluminum doping and its consequences for the physical properties of 3C-SiC. Free-standing 3C-SiC heteroepitaxial layers, intentionally doped with nitrogen or aluminum, were grown on Si (100) substrate with different 4° off-axis in a horizontal hot-wall chemical vapor deposition (CVD) reactor. The Si substrate was melted inside the CVD chamber, followed by the growth process. Micro-Raman, photoluminescence (PL) and stacking fault evaluation through molten KOH etching were performed on different doped samples. Then, the role of the doping and of the cut angle on the quality, density and length distribution of the stacking faults was studied, in order to estimate the influence of N and Al incorporation on the morphological and optical properties of the material. In particular, for both types of doping, it was observed that as the dopant concentration increased, the average length of the stacking faults (SFs) increased and their density decreased.

## 1. Introduction

Silicon carbide is a wide-bandgap semiconductor that shows high mechanical strength, chemical inertness and thermal conductivity. In particular, 3C-SiC is competitive among the SiC polytypes as it is characterized by high mobility and lower density of states at the 3C-SiC/SiO_2_ interface with respect to 4H and 6H-SiC. These properties, arising from the higher symmetry related to lower phonon scattering and lower bandgap (2.5 eV), make 3C-SiC ideal for applications in the field of power electronics [1,2] as they lead to many advantages in metal oxide semiconductor (MOS) devices as well as microelectromechanical systems (MEMS) in harsh environments [3]. Indeed, higher channel mobility and high carrier mobility imply low on-state resistance (R_ON_) for medium-voltage applications working under 1200 V, consequently lowering power dissipation in forward bias [1]. Compared to 4H and 6H hexagonal SiC, 3C-SiC films have the advantage that they can be hetero-epitaxially grown through low-temperature CVD as 3C-SiC is the most thermodynamically stable polytype. Growing a high-quality 3C-SiC epilayer on a large-area substrate would be a significant technical and scientific advancement. As a result of these features, silicon is regarded as the most intriguing substrate and crystal seed for thin epitaxial films and/or following bulk 3C-SiC growth [4]. Current technology involves the use of hetero-epitaxial growth on silicon, which inherently entails a 20% lattice parameter mismatch between Si and SiC and contributes to the generation of compressive intrinsic stress, whereas 8% at different degrees of thermal expansion provides a tensile contribution during the cooling period from the growth temperature to room temperature (RT). Such mismatches give rise to misfit dislocations and stacking faults (SFs) growing along the (111) planes of the face-centered cubic (FCC) lattice. Such defectiveness hinders the realization of devices and constitutes considerable leakage sources not compatible with the development of very large-scale integration (VLSI) technology [5].

Both nitrogen (N_2_) and ammonia (NH_3_) have been successfully employed as dopant precursors for the n-doping of SiC at temperatures above 1000 °C [6,7,8]. However, nowadays, N_2_ is the principal n-type dopant in all SiC polytypes [9]. Highly p-doped 3C-SiC is interesting for emitters in p–n-junction-based devices, because such emitters will have low resistance and can produce a high concentration of injected holes in the base region [10]. Aluminum is a preferred acceptor in SiC due to its lower activation energy (0.24 eV) compared to other acceptors (0.7 eV for B and 0.33 eV for Ga) [11]. Zielinski et al. observed that nitrogen and aluminum atoms are incorporated, respectively, on carbon and silicon sites [12].

The dopant incorporation into the SiC lattice is known to affect the crystallinity, morphology and mechanical properties of the films [13,14]. Crystallinity is very important when applying doped 3C-SiC in electronic devices because the crystal defects directly influence their leakage current and breakdown voltage.

Although 3C-SiC material for semiconductor applications has been investigated for 30 years, the problem of defect formation at the 3C-SiC/Si contact is still far from being solved. Stacking faults (SFs), partial dislocations (PDs) and anti-phase boundaries (APB) or inverted domain boundaries (IDB) are the most important defects [15]. In particular, IDBs are the main defects responsible for the electrical failure of 3C-SiC/Si-based devices [16,17]. Instead, SFs can be considered highly conducting 2D defects, in the energy range where also the bulk material is conductive. Indeed, under forward polarization, SFs were demonstrated to operate as preferred current pathways, causing a decrease in turn-on voltage [17]. In 1987, Shibahara et al. [18] observed the beneficial effect of the growth of 3C-SiC on an off-axis Si substrate with an off-axis angle from 2° to 5°. In particular, off-axis Si substrates exhibit significant improvements in the anti-phase disorder compared to on-axis ones. Moreover, off-axis growth promotes SF surface propagation lying along the (111) plane, while (−1−11) SFs opposite to the growth step are forbidden.

In this work, we present a comprehensive study of the effects of n-type and p-type doping on hetero-epitaxial 3C growth on different 4° off-axis silicon substrates (100). Micro-Raman, photoluminescence (PL) and stacking fault evaluation through molten KOH etching were performed on different doped samples in order to estimate the influence of N and Al incorporation on the morphological and optical properties of the material. The concentrations investigated in this work, 10^18^ at/cm^3^ and 10^19^ at/cm^3^ for aluminum and nitrogen, respectively, are compatible with the concentrations necessary for the development of electronic devices on 3C-SiC. In particular, for vertical metal-oxide-semiconductor field-effect transistor (MOSFET) devices, the substrate should have a concentration of approximately 10^19^ at/cm^3^ to decrease as much as possible the R_on_ of the device. Instead, for insulated gate bipolar transistors (IGBTs), the p-collector has a doping of 10^18^ at/cm^3^ [19]. Today, IGBT production is particularly expensive due to the inability to grow 4H-SiC p-doped wafers, while in the case of 3C-SiC bulk grown by CVD, it is possible to grow such types of wafers.

## 2. Materials and Methods

In this study, 3C-SiC growth was performed in a horizontal hot-wall chemical vapor deposition (CVD) reactor (ACIS M10 supplied by LPE, Catania, Italy) using (100)-oriented Si substrates. The reaction system used was tri-chloro-silane (TCS), ethylene (C_2_H_4_) and hydrogen (H_2_) as the silicon precursor, carbon precursor and gas carrier, respectively. After the growth of a 75 μm thick layer, at a temperature of 1400 °C and pressure of 100 mbar, the temperature was increased to 1650 °C and the silicon substrate was completely melted inside the CVD chamber, resulting in free-standing samples. For more details, see the references [20,21]. In particular, two different sets of samples were grown: 3C-SiC on Si (100) 4° off-axis along the [110], adding constant nitrogen flux, with a value of 0 (intrinsic), 300, 800 and 1600 sccm; 3C-SiC on Si (100) 4° off-axis along the [100], adding constant trimethylaluminum (TMA) flux, with a value of 0, 1, 2 and 4 sccm. The round brackets “()” indicate a crystallographic plane; square brackets “[]” indicate a direction. Atomic concentration values, for both types of doping, were obtained from calibration curves acquired through secondary-ion mass spectrometry (SIMS) analysis, whose values were determined to be: 1.2 × 10^19^, 2.9 × 10^19^ and 5.8 × 10^19^ at/cm^3^, for nitrogen doping; 1.7 × 10^18^ at/cm^3^, 3.4 × 10^18^ at/cm^3^ and 6.8 × 10^18^ at/cm^3^, for aluminum. In this work, two different doping scales were used, 10^18^ at/cm^3^ and 10^19^ at/cm^3^ for aluminum and nitrogen, respectively.

Micro-Raman and micro-photoluminescence maps were acquired at room temperature using an HR800 spectrometer integrated system Horiba Jobin Yvon (Horiba, Lille, France) in a backscattering configuration. For both the analyses, the excitation wavelength was supplied by a 325 nm He-Cd continuous-wave laser that was focalized on the sample by a ×40 objective, with numerical aperture (NA) of 0.5. The scattered light was dispersed by an 1800 grooves/mm kinematic grating, for Raman analysis, and by a 300 grooves/mm kinematic grating, for PL analysis.

The etching in potassium hydroxide (KOH) was adopted for the evaluation of SFs. KOH etching was performed at 500 °C for 3 min. The densities were calculated based on the observation of optical and scanning electron microscopy (SEM) images, (FE-SEM ZeissSupra35, Carl Zeiss NTS GmbH, Oberkochen, Germany) operated at an acceleration voltage of 5 kV. An accurate count and identification of SFs were achieved using powerful image analysis software.

## 3. Results and Discussion

**Figure 1:**
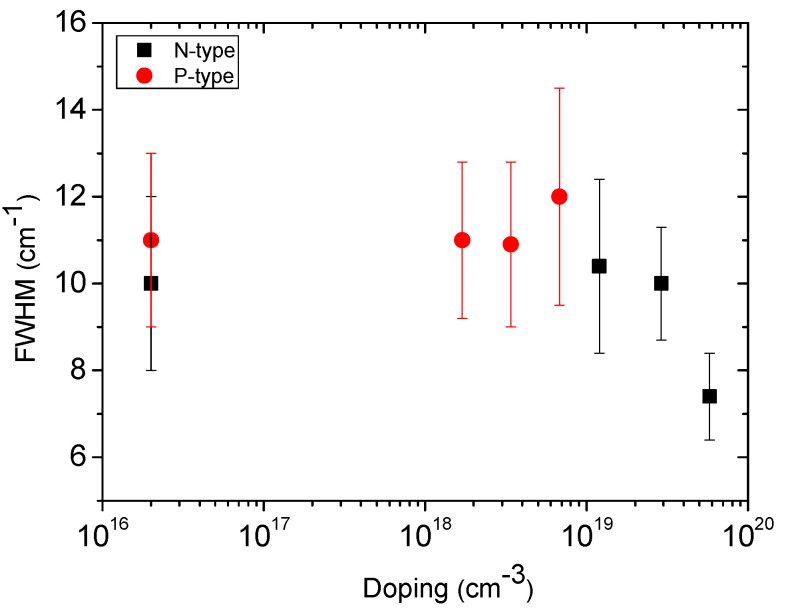
FWHM of the TO peak vs. doping concentration for nitrogen (black squares) and aluminum (red points). N-type and P-type samples were grown on Si (100) off-axis [110] and off-axis [100], respectively.

The Raman technique is sensitive to the average quality of the material. In particular, taking into account the wavelength of the laser used (325 nm), the technique is more sensitive to the surface (approximately 2–3 microns). The intensity of the transversal optical (TO) Raman mode and its full width at half maximum (FWHM) are the two parameters most closely related to the defectiveness of the material. Figure 1 shows the FWHM of the TO peak vs. doping concentration for nitrogen (black squares) and aluminum (red points). For the P-type samples, the FWHM of the TO shows a constant trend independent of the doping concentration. The FWHM value is approximately 11 cm^−1^. For the N-type samples, the FWHM of the TO decreases with increasing nitrogen concentration, reaching values lower than those of the intrinsic sample (FWHM = 10 cm^−1^) for a nitrogen concentration of 5.8 × 10^19^ at/cm^3^ (FWHM = 7 cm^−1^). However, the N-type doping is a factor of 10 greater than that of the P-type.

**Figure 2:**
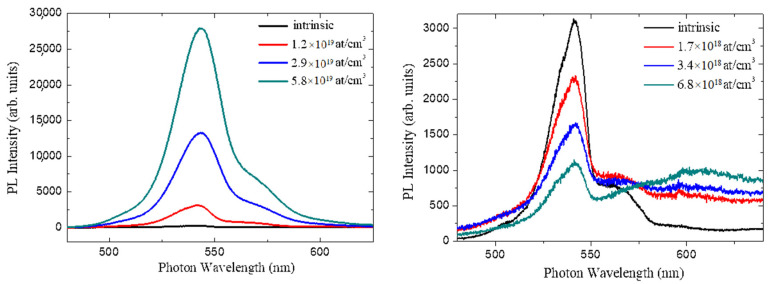
PL spectra, centered at 540 nm, acquired on the surfaces of the N-type (**left panel**) and P-type (**right panel**) samples.

Figure 2 shows the room-temperature PL spectra, centered at 540 nm, acquired on the surfaces of the N-type (left panel) and P-type (right panel) samples.

After increasing the nitrogen doping, we can observe that in the presence of N-type samples, the intensity of the band-edge peak (centered at 540 nm) grows. In N-doped 3C-SiC, there are more electrons near the Fermi level that increase the band-to-band transitions [22] and lead to the enhancement of the detected signals. The shoulder at approximately 560 nm is present in both N-type and P-type samples and it is due to the presence of levels near the conduction band introduced by nitrogen, which cannot be completely eliminated from the chamber during the growth process.

Instead, increasing the aluminum doping, in P-type samples, the intensity of the band-edge peak (centered at 540 nm) decreases. At the same time, a new band is visible in the red spectral region (approximately 600 nm), which rises in intensity when increasing the aluminum doping. In agreement with the literature [22], this band is related to the presence of intra-gap defects, which are responsible for reducing the emission intensity of the band-edge.

The presence of dopant elements not only influences the crystalline and optical properties of the material but could also influence the presence and development of SFs within 3C-SiC. Due to their high electrical activity, the concentration of SFs in the material is a fundamental parameter. Even today, the concentration of stacking faults is not compatible with the development of VLSI technology.

**Figure 3:**
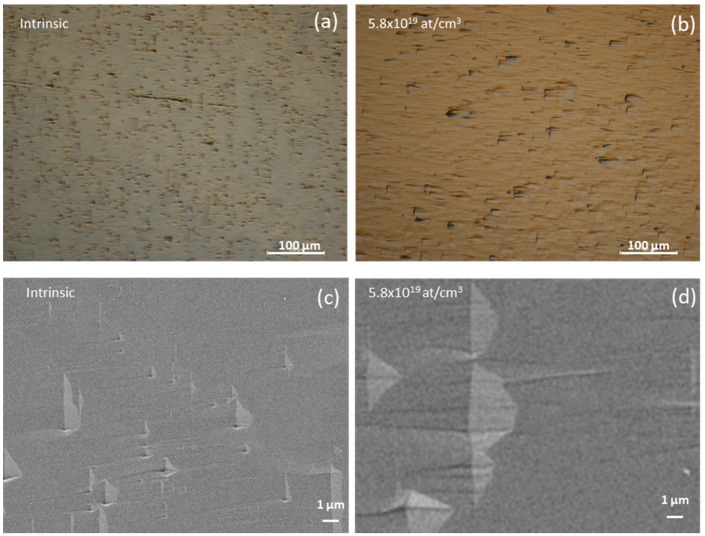
Optical and SEM images of the intrinsic (**a**,**c**) and N-type doped (**b**,**d**) samples subjected to selective KOH etching.

Therefore, all samples were treated by molten KOH etching to study the effect of doping concentration on the density and mean length of SFs. As an example, Figure 3 shows the optical and SEM images of the intrinsic (Figure 3a,c) and N-type doped (5.8 × 10^19^ at/cm^3^) samples (Figure 3b,d) subjected to selective KOH etching. Here, wet etching highlights the presence of SFs following the variation of the etching rate, which, according to the variation of binding energy in the presence of such defects, promotes groove formation along the extension of the extra planes [23].

In all images, the etched SFs are oriented along a preferential direction. Off-axis growth promotes propagation up to the surface of the SFs lying along the plane (111), while the SFs (−1−11) opposite to the growth step are forbidden. In the absence of the annihilation mechanism, the propagation of the Si-face SFs from the 3C-SiC/Si interface towards the surface is favored. In addition, the etching rate of the C-face SFs, lying on the (−111) and (1−11) crystallographic planes, is uniform to the (001) plane, while the etching rate of the Si-phase SFs is lower, displaying their distribution [24].

For the observation of the samples attached in KOH, the optical images have proved useful for the detection of large SFs (>1 µm). For SFs smaller than 1 µm, SEM images allowed us to obtain a detection limit of 100–150 nm.

**Figure 4:**
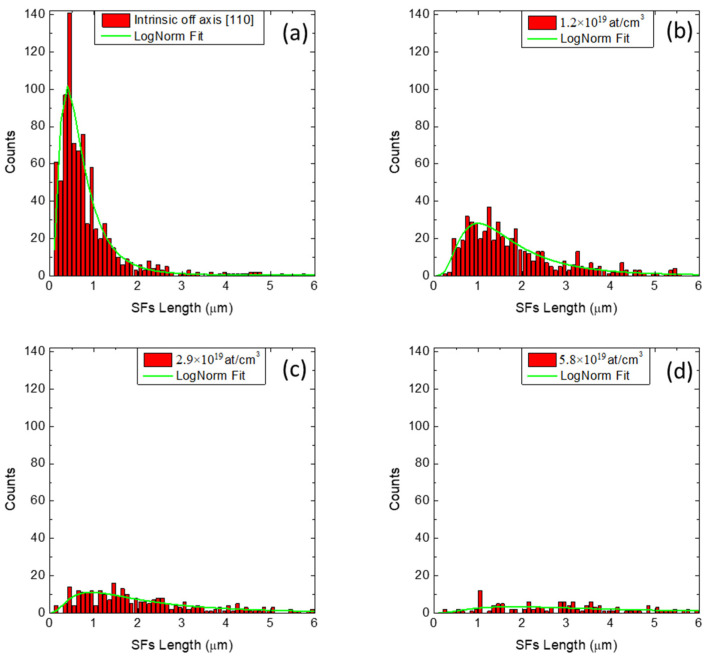
Length distribution of the SFs for different N-type doping. (**a**) Intrinsic sample, (**b**) 1.2 × 10^19^ at/cm^3^, (**c**) 2.9 × 10^19^ at/cm^3^ and (**d**) 5.8 × 10^19^ at/cm^3^. For all samples, there are a few SFs whose lengths are greater than the range reported in the X-axis range. However, all the lengths of the SFs were considered in the calculation of the mean lengths and densities.

**Figure 5:**
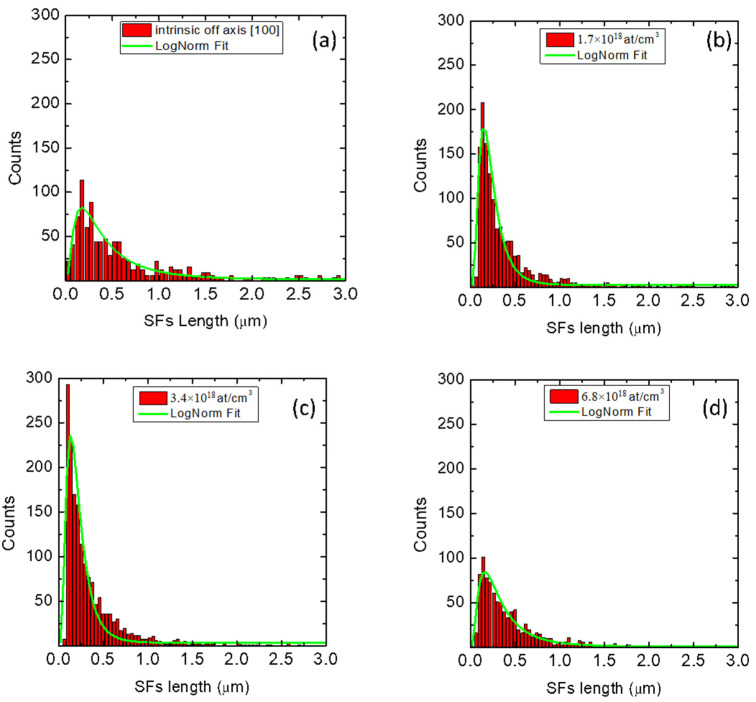
Length distribution of the SFs for different P-type doping. (**a**) Intrinsic sample, (**b**) 1.7 × 10^18^ at/cm^3^, (**c**) 3.4 × 10^18^ at/cm^3^ and (**d**) 6.8 × 10^18^ at/cm^3^. For all samples, there are a few SFs whose lengths are greater than the range reported in the X-axis range. However, all the lengths of the SFs were considered in the calculation of the mean lengths and densities.

In Figure 4 and Figure 5, we report the distribution of the lengths of the SFs for different N-type and P-type doping, respectively. For N-type samples (Figure 4a–d), we observe that as the nitrogen concentration increases (from intrinsic sample to 5.8 × 10^19^ at/cm^3^), the length distribution of the SFs widens and flattens. Consequently, the maximum shifts to greater lengths. For P-type samples (Figure 5a–d), we observe that as the aluminum concentration increases (from intrinsic sample to 6.8 × 10^18^ at/cm^3^), the maximum of the length distribution is quite constant.

**Figure 6:**
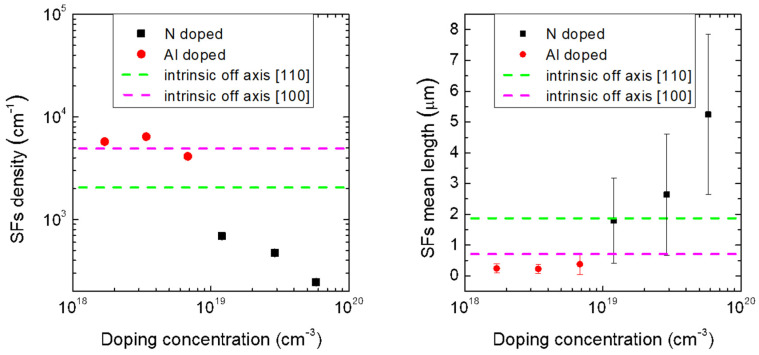
Distribution of SF density (**left panel**) and SF mean length (**right panel**) vs. doping concentration. The graphs also report the average density and average length of the intrinsic samples grown on Si (100) off-axis [110] (dashed green line) and off-axis [100] (dashed magenta line).

The distributions of the lengths of the SFs were fitted with log-normal type curves, which are better suited to fit the experimental values and to reduce the fit error. From the fits, the mean values of length of the SFs were obtained for all the samples. The values are reported in Table 1 and they are plotted in Figure 6.

We observe that by increasing the dopant concentration for P-type samples (red points, Figure 6, left panel), the mean density values of SFs are closer to the density of the intrinsic counterpart. Indeed, the Al-doped samples exhibit an SF concentration ranging from 5.74 × 10^3^ cm^−1^ for 1.7 × 10^18^ at/cm^3^ to 4.08 × 10^3^ cm^−1^ for the sample with 6.8 × 10^18^ at/cm^3^ doping. For the N-type samples (black squares, Figure 6, left panel), on the other hand, the trend of the density of the SFs tends to move away from the intrinsic value. In fact, if for the intrinsic sample, the average SF density is 2.05 × 10^3^ cm^−^^1^, by increasing doping to 2.9 × 10^19^ at/cm^3^ and 5.8 × 10^19^ at/cm^3^, such a value is notably reduced by almost an order of magnitude to 4.7 × 10^2^ cm^−^^1^ and 2.4 × 10^2^ cm^−^^1^. Ab initio DFT calculation shows that when nitrogen is very close to the SFs, their formation energy increases, resulting in a reduction in the density of the SFs inside the material [25]. Comparing the density of the SFs between the two intrinsic samples grown on Si (100) 4° off-axis along the [110] (dashed green line), and on Si (100) 4° off-axis along the [100] (dashed magenta line), two different densities were obtained: 2050 cm^−1^ and 4920 cm^−1^, respectively. The dependence of density of the SFs on the step evolution and surface morphology on the shear angle was observed for 3*C*-SiC samples grown on (111) Si substrates with off-cut axes along [110] and [112] [26]. As mentioned above, off-axis growth promotes propagation up to the surfaces of the SFs lying along the plane (111), while the SFs (−1−11) opposite to the growth step are forbidden. However, while, for the N-type samples, the off-axis angle is along the [110] direction and therefore favors the suppression of the SFs along the plane (−1−11), for the P-type samples, the off-axis angle is along the direction [100]. In this way, not all the SFs along the plane (−1−11) are suppressed. At the same time, we observed that this cutting direction does not lead to a complete suppression of the APBs (SEM images not reported here), which act as a source and annihilation point for the SFs [27]. This leads to an increase in the density of the SFs that reach the surface.

Increasing the dopant concentration for the N-type samples (black squares, Figure 6, right panel), the average length of the SFs increases, starting from a value of approximately 2 μm for the intrinsic sample to 5 μm for the sample with a nitrogen concentration of 5.8 × 10^19^ at/cm^3^. Instead, by increasing the dopant concentration for the P-type samples (red points, Figure 6, right panel), the length of the SFs remains constant, less than 1 μm. In the presence of N-type doping, the average values are greater than the value of the intrinsic sample grown off-axis [110] (dashed green line). The reduced density of SFs, increasing the nitrogen concentration (Figure 6 left panel), is susceptible to a reduced mutual annihilation mechanism. This contributes to the increase in the average size of SFs reaching the surface.

In the presence of P-type doping, instead, the average values are smaller than both values of the intrinsic samples grown off-axis (green and magenta line). As mentioned above, for the P-type samples, the off-axis angle is along the direction [100]. In this way, not all the SFs along the plane (−1−11) are suppressed. This leads to an increase in the mutual annihilation mechanism, responsible for reducing the average lengths of the SFs on the surface.

**Figure 7:**
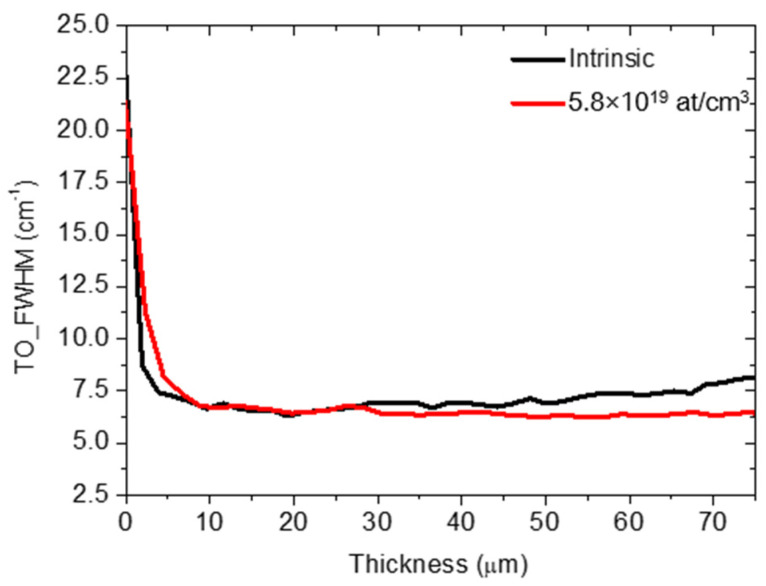
FWHM of the TO peak vs. 3C-SiC thickness in cross-section for intrinsic and 5.8 × 10^19^ at/cm^3^ (red line) samples. The zero value on the X-axis corresponds to the removed interface with the silicon.

Both values of density and mean length of the SFs depend on the direction of the cutting angle and, in particular, on the doping concentration. It is interesting to note that the trend of the density of the SFs, when increasing the doping concentration, is in agreement with the trend of the crystalline quality (FWHM of the TO) reported in Figure 1. For P-type samples, as the dopant concentration increases, a constant SF density and crystalline quality were observed. Instead, for N-type samples, as the dopant concentration increases, a clear reduction in the density of SFs and a constant increase in crystalline quality were obtained. In Figure 7, the FWHM of the TO peak vs. the 3C-SiC thickness (in cross-section) is reported, for the intrinsic (black line) and 5.8 × 10^19^at/cm^3^ (red line) samples. The zero value on the X-axis corresponds to the removed interface with the silicon.

In the first 10 µm of the films, a difference in crystalline quality was observed. In particular, lower crystalline quality was observed for the doped sample (red curve). The red curve shows a higher average value in this area. For thicknesses between 10 and 30 µm, the crystalline qualities are constant and show the same value for both samples. For thicknesses greater than 30 µm, a divergence was observed between the two curves. In particular, the doped sample continues to maintain the same crystalline quality, while for the intrinsic sample, the quality tends to worsen, reaching the value reported in the in-plan measurements (Figure 1).

The difference in thermal expansion coefficient and lattice parameters between Si and SiC leads to the formation of dislocations at the hetero-interface and the presence of a local strain field within the epilayer [15]. The presence of residual stress (depending on doping concentration [21]) can drive the formation of perfect and partial dislocations (PDs) in the epilayer. Every SF is bordered by two PDs, which limit the wrong sequence plane from the perfect 3C crystal. More investigations should be done, particularly in cross-sections, to determine the role of doping concentration in the density, shape and stability of PDs, because the expansion or shrinkage of SFs is driven by the energetics and kinetics of the PDs.

The association of current doping systems alongside compliant substrate adoption, such as inverted silicon pyramids [28] or a Si_1−x_Ge_x_ buffer layer [29], could lead to the further implementation of growth strategies capable of reducing the concentration of SFs and leading to the development of efficient 3C-SiC power devices.

## 4. Conclusions

The 3C-SiC hetero-epitaxial layers, doped with nitrogen or aluminum, were grown in a horizontal hot-wall chemical vapor deposition (CVD) reactor on Si (100) substrate with different 4° off-axis. Stacking fault evaluation through molten KOH etching was performed on different doped samples: 1.2 × 1019, 2.9 × 1019 and 5.8 × 1019 at/cm3, for nitrogen doping; 1.7 × 1018, 3.4 × 1018 and 6.8 × 1018 at/cm3, for aluminum. Samples were grown on Si (100) 4° off-axis along the [110] and on Si (100) 4° off-axis along the [100], for nitrogen and aluminum, respectively.

In particular, it was observed that as the dopant concentration increases, the average length of the SFs increases, from a value of 2 μm to 5 μm, and the density decreases, for N-type doping. Instead, for P-type doping, when increasing the doping concentration, the mean length and density of the SFs remain constant.

Both values (density and mean length) depend on the direction of the cutting angle. In fact, the off-axis angle along the [110] favors the suppression of the SFs along the plane (−1−11), decreasing the density of the SFs on the surface and increasing their mean length. Instead, for an off-axis along the [100], not all the SFs along the plane (−1−11) were suppressed. This leads to an increase in the mutual annihilation mechanism, responsible for reducing the average lengths of the SFs on the surface.

## Figures and Tables

**Table 1:** Values of average lengths and densities for different doping concentrations.

N-Type Concentration (cm^−3^)	N-Type SFs Mean Length (μm)	N-Type SFs Density (cm^−1^)	P-Type Concentration (cm^−3^)	P-Type SFs Mean Length (μm)	P-Type SFs Density (cm^−1^)
Intrinsic [110]	1.87 ± 1.12	2050 ± 19.52	Intrinsic [100]	0.71 ± 0.51	4920 ± 470
1.2 × 10^19^	1.79 ± 1.38	690.67 ± 11.40	1.7 × 10^18^	0.24 ± 0.15	5740 ± 140
2.9 × 10^19^	2.64 ± 1.97	473.78 ± 8.03	3.4 × 10^18^	0.22 ± 0.14	6360 ± 150
5.8 × 10^19^	5.24 ± 2.60	244.48 ± 10.73	6.8 × 10^18^	0.37 ± 0.33	4080 ± 100

## Data Availability

The data presented in this study are available on request from the corresponding author. The data are not publicly available due to industrial non-disclosure agreement.
